# Extracellularly Detectable Electrochemical Signals of Living Cells Originate from Metabolic Reactions

**DOI:** 10.1002/advs.202207084

**Published:** 2023-02-03

**Authors:** Kyeong‐Mo Koo, Chang‐Dae Kim, Huijung Kim, Yeon‐Woo Cho, Intan Rosalina Suhito, Tae‐Hyung Kim

**Affiliations:** ^1^ School of Integrative Engineering Chung‐Ang University Seoul 06974 Republic of Korea; ^2^ Department of Biomedical Engineering National University of Singapore Singapore 117583 Singapore

**Keywords:** drug screening, electrochemical detection, live cell sensing, metabolic reaction, stem cell senescence

## Abstract

Direct detection of cellular redox signals has shown immense potential as a novel living cell analysis tool. However, the origin of such signals remains unknown, which hinders the widespread use of electrochemical methods for cellular research. In this study, the authors found that intracellular metabolic pathways that generate adenosine triphosphate (ATP) are the main contributors to extracellularly detectable electrochemical signals. This is achieved through the detection of living cells (4,706 cells/chip, linearity: 0.985) at a linear range of 7,466–48,866. Based on this discovery, the authors demonstrated that the cellular signals detected by differential pulse voltammetry (DPV) can be rapidly amplified with a developed medium containing metabolic activator cocktails (MACs). The DPV approach combined with MAC treatment shows a remarkable performance to detect the effects of the anticancer drug CPI‐613 on cervical cancer both at a low drug concentration (2 µm) and an extremely short treatment time (1 hour). Furthermore, the senescence of mesenchymal stem cells could also be sensitively quantified using the DPV+MAC method even at a low passage number (P6). Collectively, their findings unveiled the origin of redox signals in living cells, which has important implications for the characterization of various cellular functions and behaviors using electrochemical approaches.

## Graphical abstract

This study provides novel insights into the origin of extracellularly detectable electrochemical signals of living cells. The proposed differential pulse voltammetry + metabolic activator cocktail (DPV+MAC) method, which activates key metabolic reactions in living cells, is effectively applied for rapid drug screening and non‐destructive detection of stem cell senescence.

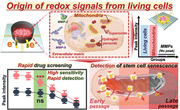

## 1 Introduction

**Figure 1:**
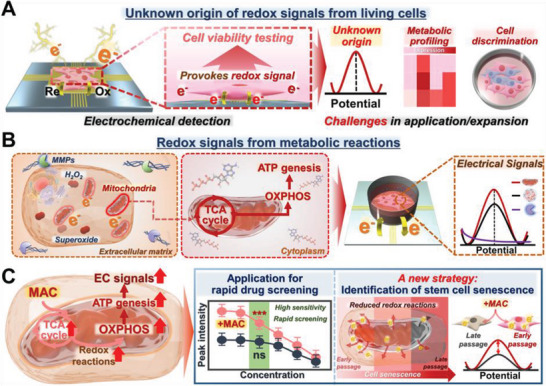
Origin of redox signals in live cells and versatile applications. A) Electrochemical methods are limited by the lack of knowledge regarding the origin of redox signals in live cells. B) Redox signals originate from cellular metabolism during metabolic reactions. C) Electrochemical methods can be used for drug screening and provide a novel means for the detection of stem cell senescence via the amplification of metabolic reactions.

Cells are complex biomachines that are responsible for all physiological functions in the human body.^[^ ^1^, ^2^, ^3^ ^]^ Therefore, analyzing their key behaviors (e.g., growth, mitosis, migration, differentiation, and death) is crucial for the discovery of novel therapeutic methods to treat various diseases, as well as to reconstruct damaged tissues or organs.^[^ ^4^, ^5^, ^6^ ^]^ One of the most frequently used methods to study such complex biochemical cascades in cells is the amplification of genes of interest (e.g., quantitative polymerase chain reaction (qPCR) and RNA sequencing (RNA‐Seq)) and tagging cell type‐specific antigens with probe‐labeled antibodies for immunocytochemistry or flow cytometry.^[^ ^7^, ^8^, ^9^, ^10^, ^11^ ^]^ Despite their proven effectiveness, these methods involve complex pretreatment steps, most of which are destructive (e.g., cell fixation and lysis) and require multiple chemical reagents or dyes, meaning that cell viability and functionality are lost after the analysis. Moreover, this approach does not allow for the real‐time detection of cellular dynamics in response to intracellular or extracellular stimuli.^[^ ^12^, ^13^, ^14^ ^]^ To overcome these limitations, new techniques such as Raman spectroscopy, autofluorescence imaging, electric cell–substrate impedance sensing (ECIS), and potentiometric analysis have been applied for the analysis of major cellular functions in a label‐free, real‐time, and noninvasive manner.^[^ ^15^, ^16^, ^17^, ^18^, ^19^, ^20^ ^]^ Among them, electrochemical methods have emerged as promising tools that enable the direct measurement of the reduction and oxidation states of target cells adhered to the surface of an electrode.^[^ ^21^, ^22^ ^]^ Specifically, previous studies have sensitively measured the viability of cancer cells grown as 2D monolayers or 3D spheroids by quantifying the intensity of redox signals from living cells.^[^ ^23^, ^24^, ^25^, ^26^, ^27^, ^28^ ^]^ Moreover, changes in the pluripotency of stem cells,^[^ ^29^, ^30^ ^]^ which generally decreases with differentiation, could be monitored without using external dyes or probes.^[^ ^31^ ^]^ However, accurate cell detection still faces several key challenges such as the maintenance of cultivation conditions, platform stability, and reproducibility. Specifically, cell analysis using these techniques cannot detect cell functions or metabolic changes. Furthermore, despite their immense potential as powerful cell analysis tools, one of the critical hurdles of electrochemical methods is that the origin of measurable redox signals in living cells is yet to be elucidated (**Figure** **1**A). To the best of our knowledge, our study is the first to report the potential sources of extracellularly detectable reduction and oxidation signals of living cells. We hypothesized that metabolic reactions to generate adenosine triphosphate (ATP), a molecule that provides energy to initiate most biological reactions in cells, are the main contributor. To prove our hypothesis, a number of possible signal contributors (i.e., electron donors or acceptors) were examined, including enzymes (e.g., matrix metalloproteinase (MMP‐2, MMP‐9), and superoxide dismutase (SOD)), redox signaling molecules (e.g., reactive oxygen species (ROS) and hydrogen peroxide (H_2_O_2_)), and mitochondria. Next, each component was isolated and further analyzed by differential pulse voltammetry (DPV) (Figure 1B). Upon confirming the detection of electrochemical signals consistent with those of living cells, major cellular metabolic pathways linked to the tricarboxylic acid (TCA) cycle and glycolysis, including oxidative phosphorylation (OXPHOS), were suppressed or enhanced to monitor the changes in the corresponding electrochemical signals. Based on our findings, we developed a method that instantaneously enhances cellular metabolism using an optimized metabolic activation cocktail (MAC) solution (Figure 1C). Finally, this method was applied for rapid and precise cancer cell‐based drug screening, as well as for label‐free and nondestructive detection of mesenchymal stem cells (MSCs) senescence, which is a major issue in the commercialization of adult stem cells.

## 2 Results and Discussion

### 2.1 Redox Signals from Living Cells Originate from Metabolic Reactions

**Figure 2:**
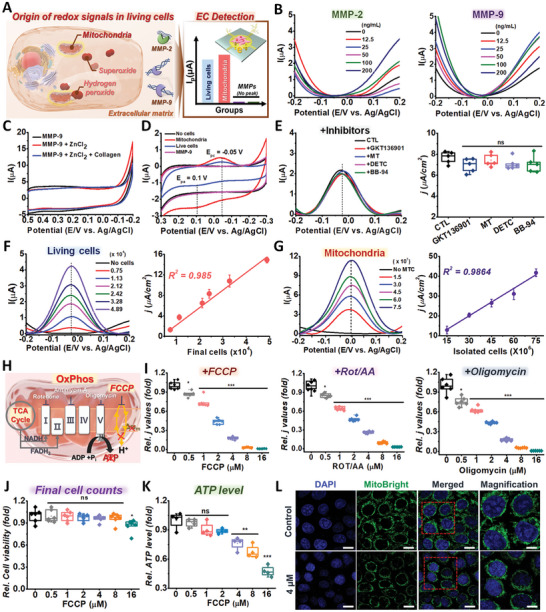
The redox signals from living cells originate from their metabolic reactions. A) Schematic diagram of the characterization of redox molecules and organelles in live cells using electrochemical methods. B) Differential pulse voltammetry (DPV) detection with varying concentrations of MMP‐2 and MMP‐9 ranging from 0 to 200 ng mL^−1^ (*n* = 3). C) Cyclic voltammetry (CV) graph of MMP‐9 (control), MMP‐9 with 10 µm of ZnCl_2_, and MMP‐9 with 10 µm of ZnCl_2_ on collagen coating platforms. D) CV signals obtained from isolated mitochondria, HeLa, and MMP‐9. E) DPV signals from HeLa cells treated with 2 µm of GKT136901, 2 µm of Mito‐Tempo (MT), 10 µm of sodium diethyldithiocarbamate (DETC), and 10 µm of batimastat (BB‐94). Box plot of the calculated current densities measured in the left panel (*j* indicates the current densities, one‐way ANOVA, *n* = 5, ***p < 0.001). F) DPV signals with varying numbers of HeLa cells ranging from 7466 to 48 866 cells. Linear correlations (*R* ^2^) of the calculated current densities from the DPV graph and final number of HeLa cells cultured on the HCGN platform (*n* = 3). G) DPV signals (*E*_p_ = −0.02 V) elicited from isolated mitochondria with various numbers of cultured HeLa (*n* = 3). Linear correlations (*R* ^2^) of the calculated current densities from the DPV graph and the mitochondria isolated from HeLa cells. H) Schematic diagram showing the mechanism of oxidative phosphorylation (OxPhos) inhibitors (rotenone (ROT) and antimycin A (AA), oligomycin, and FCCP) for HeLa cells. I) Box plot of the current densities obtained from Figure S6 of the Supporting Information (*p < 0.05, *p* < 0.001, *n* = 6). J) Final cell counting after DPV detection was performed to determine cell viability after FCCP treatment (***p < 0.001, *n* = 6). K) Total ATP levels of HeLa cells treated with various concentrations of FCCP for 24 h (**p < 0.01, ***p < 0.001, *n* = 4). L) Confocal images of HeLa cells treated with FCCP (4 µm) for 24 h and stained with MitoBright Green (green), a mitochondrial probe, with nuclear counterstaining using Hoechst 33342 (scale bar: 8 µm), and magnified images (scale bar: 3 µm).

To identify the origin of redox signals in living cells, we first focused on a biological component that is physically close to the surface of the electrode when conducting electrochemical analyses (**Figure** **2**A). Specifically, we selected matrix metalloproteinases (MMPs; i.e., enzymes responsible for the degradation of extracellular matrix (ECM) proteins) because their mechanism of action is the hydrolysis of peptide bonds (i.e., proteolytic degradation),^[^ ^32^ ^]^ which is detectable by potentiometric methods. To measure the activity of the MMPs, a highly conductive gold nanostructure (HCGN) which had been proven its stability and reproducibility, was first fabricated on indium tin oxide (ITO) (Figures S1–S3, Supporting Information) substrate via electrochemical deposition for 120 s. This material exhibited the highest sensitivity for the electrochemical signals of living cells (HeLa). MMP‐2 and MMP‐9 were separately immobilized on the fabricated HCGN at varying concentrations (0–200 ng mL^−1^) via EDC/NHS coupling and were subjected to DPV analysis (Figure 2B). We next sought to activate the MMPs by supplementing metallic ions (calcium and zinc) and collagen as a core ECM material (Figure 2C; Figure S4, Supporting Information). However, no living cell‐like signals were observed regardless of the types of electrolytes or the presence of metal ions and ECM materials. As shown in Figure 2D, no signals could be detected using cyclic voltammetry (CV), which differed from living cell signals showing a concentration‐dependent signal increase. Next, we focused on ROS because they can be easily oxidized when electrical force is applied, which could be detectable using CV or DPV. However, given that ROS are highly reactive and do not occur in their free radical forms for enough time to be detected, we sought to indirectly detect their activity using the following ROS inhibitors: 1) GKT136901, an NADPH oxidase (NOX) I/IV inhibitor to block ROS outburst, 2) Mito‐Tempo (MT), an antioxidant mixture to target mitochondria, and 3) sodium diethyldithiocarbamate (DETC), an SOD inhibitor. As shown in Figure 2E, no signal changes were observed, indicating that neither ROS generation nor their oxidation was a key mediator of intracellular redox signals.

Finally, the mitochondria, an intracellular component with its own membrane and various redox proteins that belong to the electron transport chain (ETC), was also analyzed. Remarkably, the freshly isolated mitochondria showed a linear correlation with the electrical current densities (*R* ^2^ = 0.9864), which is consistent with the signals obtained from living cells (*R* ^2^ = 0.985) (Figure 2F–G). When analyzed by CV detection, both living cells and mitochondria showed reduction and oxidation signals at the same electrical potentials at −0.05 (*E*_pc_, vs Ag/AgCl) and −0.1 (*E*_pa_, vs Ag/AgCl), respectively. The signals became nondetectable after 40 min from extraction, indicating that the metabolic activities of mitochondria are mainly responsible for the cell‐specific electrical signals (Figure S5, Supporting Information). The limit of quantification (LOQ) of our system was calculated to be 4706 cells per chip, with a linear range of 7466–48866.

After elucidating the origin of redox signals in living cells, we next sought to investigate the key contributors to the generation of electrical signals in mitochondria. We focused on the ETC because this metabolic process involves the complex oxidation and reduction of metalloproteins and related molecules. To identify the correlation between the ETC activity and electrochemical signals, oxidative phosphorylation (OXPHOS)—the major pathway for the generation of ATP in the mitochondria—was inhibited. Four different types of inhibitors were used: 1) rotenone (ROT), an ETC complex I inhibitor; 2) antimycin (AA), an ETC complex III inhibitor; 3) oligomycin, an ETC complex V inhibitor; and 4) carbonyl cyanide‐*p*‐trifluoromethoxyphenylhydrazone (FCCP), a depolarizer of mitochondrial membrane potential. Interestingly, all tested groups showed a concentration‐dependent signal decrease (Figure 2H,I; Figure S6, Supporting Information). Specifically, 4 µm ROT + AA, oligomycin, and FCCP induced 64.3%, 82.5%, and 82.2% decreases in electrochemical signals, respectively. Interestingly, no changes in cell viability were observed at the same FCCP concentration (4 µm), as determined by Prussian blue staining, whereas a significant decrease in ATP level was confirmed (23.7% decrease vs the nontreated group). These results indicate that, in addition to cell viability, the intracellular ATP level is also highly correlated with the DPV signal intensities and is thus a key contributor to the electrochemical signals of living cells. By contrast, the cell numbers remained unchanged due to the extremely short exposure of cells to ETC inhibitors (Figure 2J; Figure S6, Supporting Information). Furthermore, decreases in mitochondrial activity and corresponding ATP generation induced by low concentrations (0.5 µm) of ROT + AA, oligomycin, and FCCP were only observable through electrochemical detection (ECD) (Figure 2K; Figure S7, Supporting Information), thus demonstrating the excellent sensitivity of the DPV method over conventional colorimetric assay (Figure S8, Supporting Information). The FCCP‐induced decrease of mitochondria viability was also further confirmed via the MitoBright staining method (Figure 2I; Figure S9, Supporting Information). Taken together, our findings demonstrated that the intracellular metabolic reactions that generate ATP are the main source of redox signals in living cells, which can be sensitively detected using potentiometric methods including DPV and CV.

### 2.2 Redox Signals in Living Cells Can Be Amplified via Metabolic Activation

**Figure 3:**
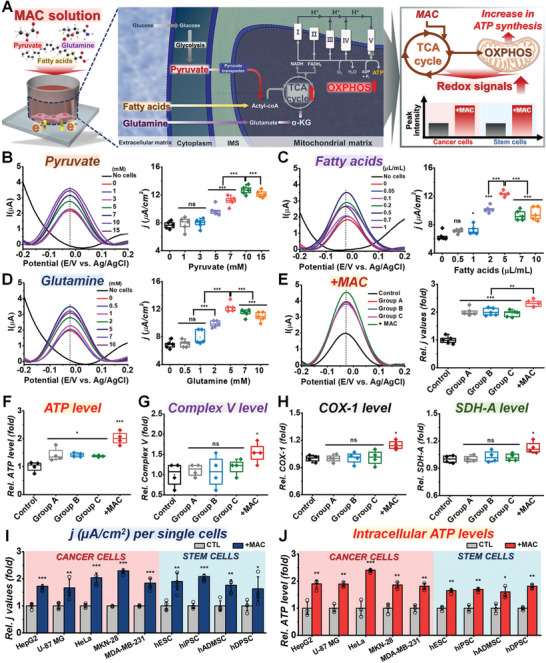
Activation of metabolic reactions results in redox signal amplification. A) Schematic diagram showing the mechanism of pyruvate‐, fatty acid‐, and glutamine‐induced metabolic activation in cancer/stem cells and their redox signals. B) Optimization of pyruvate concentrations for amplification of electrochemical signals in HeLa cells. The calculated current densities obtained from the DPV graph are presented as a box plot (*p < 0.05, ***p < 0.001, *n* = 5). C) Optimization of fatty acids according to their concentrations and their electrochemical signals. The calculated current densities from the DPV graph are presented as a box plot (*p < 0.05, ***p < 0.001, *n* = 5). D) DPV graph of optimization for glutamine at various concentrations. The DPV signals obtained from the DPV graph are shown as a box plot (***p < 0.001, *n* = 5). E) DPV signals of HeLa cells treated with various combinations of metabolic activators (optimized concentrations for each compound) including Group A (pyruvate and glutamine), Group B (pyruvate and fatty acids), Group C (fatty acids and glutamine), and MAC for 90 min. The current densities obtained from the DPV graph are presented as a box plot (**p < 0.01, ***p < 0.001, *n* = 5). F) Quantification of ATP level from HeLa cells treated with various combinations of metabolic activators for 90 min, presented as a box plot (*p < 0.05, ***p < 0.001, *n* = 4). G) Quantification of the mitochondrial respiratory complex V activity of HeLa cells treated with various combinations of metabolic activators (*p < 0.05, *n* = 4). H) ELISA of the levels of mitochondrial proteins, COX1, and SDH‐A in HeLa cells treated with various combinations of metabolic activators for 90 min (*p < 0.05, *n* = 4). I) Current densities obtained from Figure S11 and Table S1 of the Supporting Information, presented as a bar graph (*p < 0.05, **p < 0.01, ***p < 0.001, *n* = 4). J) Intracellular ATP levels from MAC‐treated HeLa cells, presented as a bar graph (*p < 0.05, **p < 0.01, ***p < 0.001, *n* = 4).

Given that intracellular metabolic reactions to produce ATP were found to be the main origin of the redox signals in living cells, we next sought to investigate whether these signals can be amplified through external supplementation of metabolic intermediates (**Figure** **3**A). We first supplemented the cells with pyruvate (the end product of glycolysis) because it is a key molecule for the synthesis of citrate^[^ ^33^ ^]^ (the first metabolite of the TCA cycle) from oxaloacetate and acetyl‐CoA with the help of pyruvate dehydrogenase (PDH).^[^ ^34^ ^]^ As shown in Figure 3B, electrochemical signals were enhanced with increasing pyruvate concentrations. Based on this finding, we added other types of metabolic intermediates, including fatty acids as acetyl‐CoA precursors and glutamine to accelerate alpha‐ketoglutarate (*α*‐KG) synthesis.^[^ ^35^ ^]^ All of these components resulted in statistically meaningful increases in electrochemical signals in a concentration‐dependent manner. Specifically, signals were enhanced by ≈65.2%, 92.9%, and 78.4% when treated with 10 mm pyruvate, 5 µL mL^−1^ fatty acids, and 5 mm glutamine, respectively, compared to the control (nontreated) groups (Figure 3C,D). However, when the concentration exceeded these specific levels, the EC signals decreased due to ROS‐mediated cellular damage. Based on this interesting finding, we next mixed all of these components to synergistically amplify ATP synthesis, which ultimately enhanced the electrochemical signals of living cells. The DPV detection of living cells was performed after treating the cells with media containing the following metabolic enhancers: 1) pyruvate and fatty acids (Group A), 2) pyruvate and glutamine (Group B), 3) fatty acids and glutamine (Group C), and 4) pyruvate, fatty acids, and glutamine (MAC). The media treatment time was fixed at 90 min for all experimental conditions based on the optimization results (Figure S10, Supporting Information). As shown in Figure 3E, all four groups exhibited a significant increase in current densities. Specifically, groups A to C lacking one of the metabolite intermediates exhibited 195–202% increases, whereas the MAC group containing all three components showed a 231.2% increase in DPV signals. Consistent with the results of inhibiting ETC proteins (Figure 2), the cells treated with metabolic enhancer‐containing media exhibited enhanced ATP levels (Figure 3F), further supporting the strong correlation between electrochemical signal intensities and intracellular ATP levels. No changes in the expression levels of proteins involved in ETC were found, including succinate dehydrogenase complex‐subunit A (SDH‐A, ETC complex II), cytochrome *c* oxidase I (COX‐1, ETC complex IV), and ATP synthase (complex V) (Figure 3G,H). These results indicated that adding metabolic enhancers does not affect the downstream or epigenetic regulation of ETC‐related protein expression due to the short treatment time. We next applied this MAC solution to other types of cells, including cancer (e.g., liver cancer, glioblastoma, cervical cancer, stomach adenocarcinoma, and breast cancer) and stem cells (e.g., embryonic stem cells, induced pluripotent stem cells, and mesenchymal stem cells) (Figure S11, Supporting Information). As shown in Figure 3I,J and Table S1 (Supporting Information), the MAC‐treated groups exhibited 1.63–2.2‐fold increases in current densities and intracellular ATP levels regardless of cell type, thus demonstrating the wide application potential of the proposed DPV + MAC method.

### 2.3 Application for Rapid Anticancer Drug Screening Using the DPV + MAC Method

**Figure 4:**
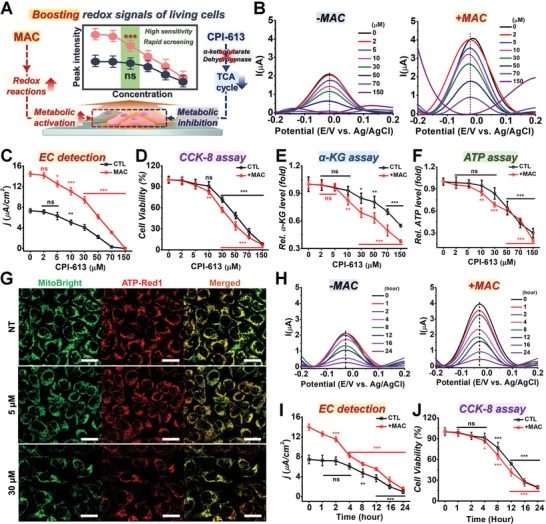
Application for rapid anticancer drug screening. A) Schematic diagram showing the amplification of redox signals from living cells after CPI‐613 treatment. B) DPV signals from HeLa cells exposed to different concentrations of CPI‐613 for 24 h without MAC (left panel) and with MAC (right panel), ranging from 0 to 150 µm. C) Current densities calculated from (B), presented as a line graph, the black and red lines represent the control and MAC treatments, respectively (*p < 0.05, **p < 0.01, ***p < 0.001, *n* = 3). D) CCK‐8 results from HeLa cells exposed to various concentrations of CPI‐613 with/without MAC (**p < 0.01, ***p < 0.001, *n* = 3). E,F) *α*‐Ketoglutarate (D) and intracellular ATP levels of HeLa cells exposed to different concentrations of CPI‐613. G) Representative immunostained images of mitochondrial membrane (MitoBright) and ATP levels (ATP‐Red1) in HeLa treated with various concentrations of CPI‐613. H) DPV graph from HeLa cells treated with 70 µm of CPI‐613 for 24 h without MAC (left panel) and with MAC (right panel). I) Current densities of DPV results from HeLa cells from (H), presented as line graphs for 24 h (*p < 0.05, **p < 0.01, ***p < 0.001, *n* = 4). J) Calculated live cell percentages based on the CCK‐8 results from HeLa cells exposed to 70 µm of CPI‐613 for 24 h (*p < 0.05, **p < 0.01, ***p < 0.001, *n* = 4).

After confirming a method to enhance the electrochemical signals of living cells via instantaneous amplification of metabolic activity, we next sought to confirm the applicability of electrochemical techniques for in vitro drug screening and stem cell characterization. First, CPI‐613, a molecule that inhibits the activities of PDH and *α*‐ketoglutarate dehydrogenase, was applied to cervical cancer cells.^[^ ^36^, ^37^ ^]^ After the drug treatment, the cells were treated with MAC solution for 90 min to amplify the DPV signals (**Figure** **4**A). As shown in Figure 4B, the MAC solution‐treated groups showed 1.982.76‐fold stronger signals at a CPI‐613 concentration range of 270 µM. These signal differences between the control and MAC‐treated groups were not detectable with *α*‐KG, ATP assays, immunostaining (MitoBright and ATP‐Red 1), or a conventional colorimetric assay (CCK‐8), indicating that the proposed DPV + MAC method had an extremely high sensitivity (Figure 4C–G; Figure S12, Supporting Information). Specifically, a decrease in current densities was observed at 5 µm of CPI‐613, whereas the control (CTL, without MAC) group failed to show statistical significance at the same concentration (Figure 4C). All other analytical results, including colorimetric cell viability (CCK‐8), intracellular ATP, and *α*‐KG levels, showed statistical differences at CPI‐613 concentrations higher than 10 µm even with MAC treatment (Figure 4D–G). Given the extremely high sensitivity of the DPV + MAC method for the detection of intracellular metabolic activities, we next conducted a pharmacokinetic study. As shown in Figure 4H,I, the immediate changes in electrochemical signals 1–4 h after the CPI‐613 treatments were only observable in DPV + MAC group. These differences were not measurable with the CCK‐8 assay after up to 4 h of drug treatment (Figure 4J). Moreover, the detection was extremely rapid (<30 s) and did not require any chemical reagents for the analysis.

### 2.4 Nondestructive Detection of Stem Cell Senescence and Proliferation Using the DPV + MAC Method

**Figure 5:**
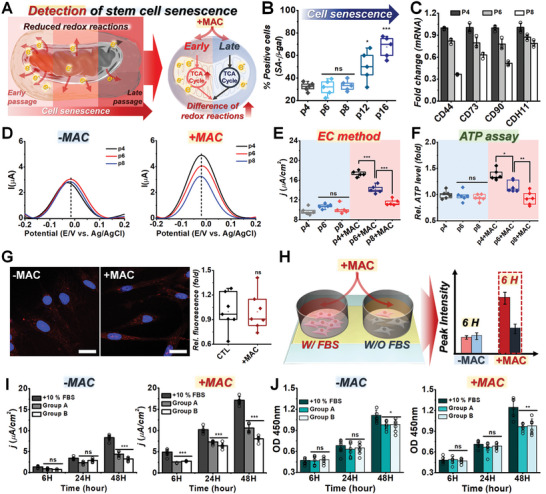
Nondestructive detection of stem cell senescence and proliferation. A) Schematic illustration depicting a new strategy for the detection of stem cell senescence. B) Stained levels of SA‐*β*‐gal‐positive cells from Figure S14 of the Supporting Information, digitized for analysis with the ImageJ software (*p < 0.05, ***p < 0.001, *n* = 5). C) Gene expression results of human adipose‐derived mesenchymal stem cells (hADMSCs) at several passages; the data were normalized to the control (P4) (*p < 0.05, **p < 0.01, ***p < 0.001, *n* = 3). D) DPV signals obtained from various passages without MAC treatment (left panel) and with MAC treatment (right panel). E) Calculated current densities observed in (D) presented as a box plot (one‐way ANOVA, *n* = 5, ***p < 0.001). F) ATP assay results of hADMSCs at several passages; the data were normalized to the control (P4) (*p < 0.05, **p < 0.01, *n* = 6). G) Representative confocal images of hADMSCs (P5) stained with MitoSox (red) and Hoechst 33342 (blue). Quantification of MitoSox‐positive cells from (left‐panel), digitized for analysis with the ImageJ software (one‐way ANOVA, ns *p* > 0.05, *n* = 7). H) Schematic illustration of the early detection of stem cell proliferation using the EC method. I) Comparison of the DPV signals (current densities) of hADMSCs between the −MAC (left panel) and +MAC (right panel) groups over the course of 48 h under different medium conditions including a control group (commercial culture medium for MSCs), Group A (5% FBS), and Group B (low glucose, and no FBS) obtained from Figure S15 of the Supporting Information (***p < 0.001, *n* = 6). J) Cell proliferation results between the −MAC (left panel) and +MAC (right panel) groups over the course of 48 h under different medium compositions (*p < 0.05, **p < 0.01, *n* = 6).

Next, we evaluated the applicability of the DPV + MAC method for stem cell research. It has been reported that adult stem cells, including mesenchymal stem cells (MSCs), experience senescence with passaging, which critically affects in vitro differentiation and further tissue regeneration efficiency when applied for clinical trials.^[^ ^38^, ^39^ ^]^ The senescence‐associated *β*‐galactosidase (SA‐*β*‐gal) staining method is currently the gold standard for senescence assessment in vitro. However, this method requires expensive external staining dyes and multiple treatment steps. Therefore, we sought to electrochemically detect MSC senescence, which is directly correlated with metabolic activities (**Figure** **5**A). As shown in Figure 5B and Figure S13 (Supporting Information), senescence‐associated *β*‐galactosidase (SA‐*β*‐gal) positive cells were gradually increased with passing, especially after P12. The median value for the percentage of SA‐*β*‐gal‐positive cells among P16 MSCs was ≈67.5%, which was 35.2% higher than that of P4 MSCs. Moreover, the MSC multipotency‐specific gene and protein expressions were found to decrease at low passage numbers (<P8) but were nondetectable with the SA‐*β*‐gal assay (Figure 5C; Figure S14A, Supporting Information). Additionally, as shown in Figure 5D, no signal differences were found between the P4, P6, and P8 groups. However, when treated with MAC for 90 min, the DPV signals decreased with passaging (Figure 5E). Specifically, the DPV signals of MSCs at P6 and P8 were only 18.1% and 33.5% relative to the signal intensity of P4 MSCs, respectively (*N* = 5). We also confirmed 15.9% and 16.4% decreases in ATP levels in MAC‐treated MSCs at P6 and P8, respectively, demonstrating that there was a high correlation between the electrochemical signals and cellular metabolic activities (Figure 5F). Furthermore, our experiments with different MSC multipotency markers (e.g., CD73, CD90, and CD105) demonstrated that MAC treatment had no adverse effects on cell viability (i.e., mitochondrial dysfunctions) and multipotency, which is adversely affected by ROS (Figure 5G; Figure S14B,C, Supporting Information).

In addition to senescence, one of the most important topics in MSC research is the optimization and development of cultivation media. There are currently more than 98 companies worldwide that commercialize MSC medium as a complete kit.^[^ ^40^ ^]^ The goals of medium development are to 1) accelerate MSC growth and maintain MSC multipotency and 2) avoid the use of unknown or animal‐derived growth factors. Therefore, in addition to the detection of stem cell senescence, methods to assess medium performance within a short incubation time are also urgently needed.^[^ ^41^ ^]^ Remarkably, as shown in Figure 5H, the differences in cell growth potential between full growth factor (10% FBS), reduced growth factor (Group A, 5% FBS), and no growth factor with low glucose (Group B) were detectable within only 6 h of incubation using the proposed DPV + MAC method (Figure 5I; Figure S14, Supporting Information). These differences could not be detected using conventional colorimetric assays in either the −MAC or +MAC groups even after 24 h of incubation (Figure 5J). After 48 h, the DPV peak intensities for Group A and Group B became 1.85 and 2.56 µA, respectively, and were statistically distinguishable (*p* < 0.005) even without MAC treatment, which could not be assessed with the CCK‐8 assay. Taken together, our findings demonstrated that our proposed electrochemical detection method coupled with MAC treatment could be applied to various cellular studies, including rapid anticancer drug screening, stem cell senescence detection, and the evaluation of cell culture media performance, and is thus an immensely valuable tool.

## 3 Conclusion

Our study elucidated the origin of redox signals in living cells, which are detectable using voltammetric methods. Mitochondria immobilized on an HCGN electrode exhibited concentration‐dependent electrochemical signals, which were matched with cellular signals. By sequentially inhibiting mitochondrial membrane proteins (e.g., Complex I, III, V, and ATP synthase) belonging to the ETC, our findings demonstrated that the redox signals in living cells originated from intracellular ATP levels and related mitochondrial redox reactions. Based on these findings, we next attempted to amplify the electrical signals by controlling key metabolic pathways. A MAC solution consisting of pyruvate, fatty acids, and glutamine at different concentrations induced 1.47–2.3‐fold increases in signal intensity regardless of cell type, from cancer to stem cell lines, even after an extremely short treatment time (90 min). Due to the high sensitivity of our proposed approach, the effects of CPI‐613 on cervical cancer cells could be detected even at low drug concentrations (2 µm) and an extremely short incubation time (1 h). Moreover, our proposed DPV + MAC method could also be applied to assess the senescence of human MSCs (hMSCs), which was not measurable using conventional SA‐*β*‐gal staining, especially at an early passage (<P6).

Recently, numerous large pharmaceutical companies have sought to develop new types of drugs capable of selectively disrupting mitochondrial activities and cancer cell functions.^[^ ^42^, ^43^ ^]^ In the case of stem cell research, there is a pressing need for novel techniques capable of quantifying multipotency, differentiation, and function in a label‐free and nondestructive manner. Given that such cellular activities are highly correlated with metabolic reactions, our discovery of the origin of cellular redox signals and our proposed DPV + MAC approach will be highly impactful not only for developing novel anticancer drugs but also for quality control or standardization of stem cell‐based therapeutic products.

## 4 Experimental Section

### Materials

An indium tin oxide (ITO) electrode was obtained from U.I.D. (Cheongju, South Korea). Gold(III) chloride trihydrate (HAuCl_4_), poly(ethylene glycol) 200 (PEG 200), Triton X‐100, and phosphate‐buffered saline (PBS) were purchased from Sigma‐Aldrich (Saint Louis, Missouri, USA). Polydimethylsiloxane (PDMS) was obtained from Dow Corning Crop. (Midland, MI, USA). The solutions used in this study were prepared with deionized (DI) water, which was purified using a Millipore Milli‐Q Direct Water Purification System (EMD Millipore, MA, USA).

### Fabrication and Characterization of the HCGN Platform

The ITO electrode was first cleaned using an ultrasonic bath for 20 min with Triton X‐100, DI water, and 70% ethanol (EtOH). A plastic chamber with a 0.6 cm diameter was affixed to the ITO electrode using bio‐friendly glue (PDMS) to allow for the electrochemical deposition of the gold mixture solution and further maintain the cell growth on a platform. The gold mixture solution was composed of 5 mm gold(III) chloride solution with PEG at a ratio of 50:1 for electrochemical detection. The electrochemical deposition of the gold mixture solution on the ITO electrode was performed using the multistep potential channel with various deposition times (0, 30, 60, 90, 120, 130, 140, 150, and 160 s). Electrochemical deposition was performed at room temperature (RT). The calculated total electrode area was 0.283 cm^2^. Then, the ready‐to‐use cell chip was washed using 70% ethanol and 1× DPBS. The cell chip was sterilized under UV light for 1 h to prevent contamination. After sterilization, the HCGN platform was coated with various coating materials (e.g., Geltrex LDEV‐free reduced‐growth‐factor basement membrane matrix, Matrigel, and human plasma fibronectin) for the cultivation of pluripotent stem cells and incubated for 1 or 1.5 h depending on the manufacturer's guidelines (37 °C, 5% of CO_2_). Before cell seeding, the coated cell chip was washed with cultured media.

To characterize the surface of the HCGN platform, field‐emission scanning electronic microscopy (FE‐SEM, Sigma Family, Carl Zeiss, Germany), and atomic force microscopy (AFM, XE‐100, PSIA, South Korea) with a noncontact mode was utilized. Before conducting the FE‐SEM and AFM analyses, the fabricated platform was washed with UltraPure DNase/RNase‐Free Distilled Water (Invitrogen, USA) and dried at RT to ensure that the surface of the platform was clean.

### Cell Culture and Cultivation

Human glioblastoma cells (U‐87MG), liver cancer cells (HepG2), gastric cancer cells (MKN‐28), and breast cancer cells (MDA‐MB‐231) were obtained from the Korean Cell Line Bank. Additionally, cervical cancer cells (HeLa) were obtained from the American Type Culture Collection (ATCC, Manassas, VA). hESCs (H9, passage 90–92), human induced pluripotent stem cells (hiPSC CMC11), and human adipose tissue‐derived MSCs (hADMSCs) were acquired from the Wicell Research Institute, the Catholic University, and Cell Engineering For Origin (CEFO Co., Ltd., Seoul, South Korea), respectively. Human dental pulp stem cells (hDPSCs) and culture media were also purchased from Lonza (Lonza Group Ltd., Basel, Switzerland). U‐87MG, MDA‐MB‐231, and HeLa cells were maintained in Dulbecco's modified Eagle medium (DMEM) (Thermo‐Fisher, Germany) supplemented with 10% foetal bovine serum (FBS) and 1% antibiotic–antimycotic at 37 °C with 5% CO_2_. HepG2 cells and MKN‐28 were cultured in MEM and RPMI1640 medium supplemented with 10% FBS and 1% antibiotic–antimycotic (Thermo‐Fisher, Germany), respectively. The mTeSR1 media (Stem Cell Technologies, Canada) was used for hESCs and hiPSC cultivation. Particularly, both cell lines were required for cultivation in 6‐well plates coated with Matrigel diluted in DMEM/F12 (Thermo‐Fisher, Germany) at a 1:80 ratio (BD Biosciences, CA, USA) and 1% Geltrex (Gibco, USA), respectively. Before cell plating, the pellet of each cell line was washed with mTeSR1 media containing 10 µm Y‐27632 (Rho‐kinases [ROCK]) inhibitor (Tocris) to maintain the cell viability. The growth media was exchanged every 24 h for proliferation. For maintenance of hADMSCs and hDPSCs, the cells were grown in growth media supplemented with 10% FBS (CEFO) and 1% antibiotic–antimycotic (Gibco, Seoul, South Korea). The hADMSCs and hDPSCs were grown on 100‐mm tissue culture dishes coated with human plasma fibronectin (Gibco, Seoul, South Korea, 1:40 dilution) at 37 °C for 1 h. For DPV detection, various cancer cells on the chip including HeLa, MKN‐28, U‐87 MG, Hep G2, and MDA‐MB‐231 were detached with 0.25% Trypsin‐EDTA solution (Gibco, Seoul, South Korea). Mesenchymal stem cells such as hADMSCs and hDPSCs were detached with 1× TrypLE (Gibco, Seoul, South Korea). Pluripotent stem cells such as hiPSCs and hESCs were detached using ReLeSR (Stem Cell Technologies, Canada). All of the cell lines were grown and processed according to standardized protocols. After DPV detection, the electrochemically detected cells on the HCGN platform were detached using a 0.25% Trypsin–EDTA solution and counted with a hemocytometer using the following formula

(1) $$\begin{array}{c} \begin{array}{c} \mathrm{Total}\;\mathrm{cells}\;\left(mL\right)=\mathrm{total}\;cells\;counted\times \frac{\mathrm{dilution}\;\mathrm{factor}}{4\;\mathrm{of}\;\mathrm{squares}}\times 10\;000\;\mathrm{cells}\;{\mathrm{mL}}^{-1} \end{array} \end{array}$$

### Optimization of MAC for Boosting Redox Signals

The conditions of the MAC were optimized using sodium pyruvate (Gibco, Seoul, South Korea), l‐glutamine solution, and fatty acids (Merck Millipore, MA, USA). When optimizing and mixing components, cultured media was used as a solvent to maintain the functions, morphologies, and pH conditions of the cells. Before exchanging from cultured media to MAC onto the HCGN platform, the fabricated chip was washed with DPBS. Then, the optimized combination was added to the chip and incubated for 90 min at 37 °C. After incubation, MAC was removed, and fresh cultured medium was added to eliminate any residual redox molecules for electrochemical detection.

### ECD of Living Cells

CV and DPV were performed using a DY2000 series multichannel potentiostat (Digi‐Ivy). Ag/AgCl (1 m KCl) and platinum wires were used as reference and counter electrodes, while the fabricated platform served as a working electrode. For ECD analysis of live cells, each growth medium for the various cell lines was washed with 1× DPBS before cell detection. CV was performed under the following parameters: initial *E* (V) = 0.3; high *E* (V) = 0.3; low *E* (V) = −0.3; scan rate (V) = 0.05. DPV signals were detected under the following conditions: initial *E* (V) = −0.3; final *E* (V) = 0.4; step *E* (V) = 0.005; sampling time (s) = 0.02; pulse period (s) = 0.2. All electrochemical detections were conducted at RT. All cultured media was exchanged before the ECD analyses to prevent any potential signal interferences from the cultured cells during cultivation. After exchanging the culture media, the cells cultured on the chips were incubated for 90 min. From the DPV signals, the current density was calculated based on the following formula

(2) $$\begin{array}{c} \begin{array}{c} j\left(\mathrm{current}\;\mathrm{density}\right)=I/A\left(I:\mathrm{measured}\;\mathrm{current}\;\mathrm{values},\;A:\mathrm{area}\;\mathrm{of}\;\mathrm{electrode}\right) \end{array} \end{array}$$

Then, the calculated current densities (*j*) were determined by subtracting the baseline current from the current density of DPV at *E*_p_ = 0 V. After the DPV detection, the LOD and LOQ from the DPV results were calculated with the following two formulas

(3) $$\mathrm{Limit}\;\mathrm{of}\;\mathrm{detection}\;\left(\mathrm{LOD}\right)=\frac{3.3\sigma }{S}\left(\sigma :\mathrm{standard}\;\mathrm{error},\;S:\mathrm{slope}\right)$$

(4) $$\mathrm{Limit}\;\mathrm{of}\;\mathrm{quantification}\left(\mathrm{LOQ}\right)=\frac{10\sigma }{S}\left(\sigma :\mathrm{standard}\;\mathrm{error},\;S:\mathrm{slope}\right)$$

### ECD of MMPs

CV and DPV were conducted using a DY2000 device. MMP‐2 and MMP‐9, which are widely used for research,^[^ ^44^ ^]^ were obtained from Sigma‐Aldrich (MO, USA). To activate the MMPs, collagen and zinc chloride were purchased from Sigma‐Aldrich. For MMP detection, various solvents were first electrochemically detected using CV. CV detection was performed as follows: initial *E* (V) = 0.8; high *E* (V) = 0.8; low *E* (V) = −0.2; scan rate (V) = 0.05. Particularly, before ECD, each sample was washed with 1× DPBS, which was the solvent used to suspend the MMPs. Then, CV was conducted as follows: initial *E* (V) = 0.5; high *E* (V) = 0.5; low *E* (V) = −0.3; scan rate (V) = 0.05. DPV detection was performed under the following conditions: initial *E* (V) = −0.3; final *E* (V) = 0.4; step *E* (V) = 0.005; sampling time (s) = 0.02; pulse period (s) = 0.2. All electrochemical detections were conducted at RT. MMPs were incubated for 24 h at 37 °C to react with zinc chloride or collagen, as these proteins are known to degrade collagen^[^ ^45^ ^]^ and combine with zinc ions.

### Isolation and Electrochemical Detection of Mitochondrial Signals

To monitor the mitochondrial redox signals, cultured HeLa cells were isolated using a commercial mitochondria isolation kit (Thermo‐Fisher, Germany). To prevent damage to mitochondrial membranes and functions (e.g., ATP genesis and redox reactions), all procedures were conducted as described in previous studies. CV was conducted under the same conditions as EC detection of live cells. DPV was performed as follows: initial *E* (V) = −0.3; final *E* (V) = 0.4; step *E* (V) = 0.005; sampling time (s) = 0.02; pulse period (s) = 0.2. To maintain the mitochondrial redox reactions and outer membrane potential activity, the EC detection of mitochondria was rapidly performed within 15 min in trehalose buffer as previously reported.^[^ ^46^ ^]^

### Assessment of Various Inhibitors and CPI‐613 Treatment

To investigate the origin of redox signals, various inhibitors such as sodium diethyldithiocarbamate, GKT136901 (Sigma‐Aldrich, MO, USA), Mito‐tempo (MedChemExpress, NJ, USA), FCCP, oligomycin, rotenone and antimycin A (Agilent, CA, USA), and Batimastat (Selleckchem, Seoul, South Korea) were used as inhibitors. All of the inhibitors were incubated for 24 h at 37 °C. Furthermore, the cells were treated with CPI‐613 (MedChemExpress, NJ, USA) for drug screening and incubated for 24 h at 37 °C. After treatment, the cells were washed with DPBS for EC detection within 30 s and cytotoxicity testing.

### Measurements of Total Cellular ATP Level

An ATP assay kit (ab83355, Abcam, UK) was used following the manufacturer's instructions to detect the total ATP level from the whole cells as described in a previous study.^[^ ^47^ ^]^ Prior to measuring the ATP levels, 1 × 10^6^ of cultured HeLa cells were harvested from the fabricated platform. For ATP level detection, ATP standards of 50 µL (labeled from 1 to 6) were prepared to construct a standard curve. Next, the harvested samples were centrifuged at 13 000 × *g* for 5 min at 4 °C to remove any impurities that could have interfered with the analyses. The supernatants were collected and then transferred to new tubes. The volume of all samples was adjusted to 50 µL with ATP assay buffer. Then, 50 µL of the reaction mixture was added to the background sample wells. After conducting all of the procedures, the loaded 96‐well plate was incubated at room temperature for 30 min protected from light. The absorbance was measured using a microplate reader (Synergy H1 Hybrid Reader, BioTek, VT, USA) at a 570 nm wavelength. The experiment was repeated at least three times.

### Measurements of Intracellular Levels of *α*‐Ketoglutarate

*α*‐Ketoglutarate levels were measured using an *α*‐ketoglutarate assay kit (ab83431, Abcam, UK). Samples preparation and the *α*‐ketoglutarate assay were conducted according to the manufacturer's instructions. Lysates of HeLa cells were incubated with an enzyme reaction mixture for 30 min at 37 °C for transamination of *α*‐ketoglutarate, after which the generated pyruvate reacted with a probe that generated a specific color. The samples were then measured using a microplate reader (Synergy H1 Hybrid Reader, BioTek, VT, USA) at 570 nm. Finally, the concentration of *α*‐ketoglutarate was calculated using a standard curve.

### Measurement of Mitochondrial Proteins Level

HeLa cells cultured on the HCGN platform were treated with MAC for 90 min. The HeLa cells were then fixed with 4% paraformaldehyde. Mitochondrial biogenesis was measured using the colorimetric Mitobiogenesis In‐Cell ELISA kit (ab119217, Abcam, UK), which simultaneously quantifies the levels of mitochondrial DNA‐encoded cytochrome *c* oxidase I (COX‐1) and nuclear DNA‐encoded succinate dehydrogenase A (SDH‐A) within the HeLa cells. The colorimetric assay was performed in a 96‐well plate format. All experiments were performed according to the manufacturer's instructions, as previously reported.

### Visualization of the Mitochondrial ATP of Living Cells via Fluorescence Imaging

The BioTracker ATP‐Red dye (Millipore) functions as live cell imaging for cellular ATP localized in the mitochondria. The probe is nonfluorescent when forming a closed ring structure. In the presence of negatively charged ATP, the covalent bonds between boron and ribose are broken and the ring opens producing fluorescence. ATP red staining was conducted by immersing the samples in culture medium containing 5 µm ATP red dye, cells were incubated for 15 min at 37 °C in a humidified atmosphere containing 5% CO_2_. Cells were washed twice with DPBS prior to the addition of fresh medium. All fluorescence images were visualized using a Leica DMi8 confocal microscope (Leica, Germany). All procedures were performed as described in a previous study.^[^ ^48^ ^]^

### Live Imaging of Mitochondrial Membrane Potential Activity and mtROS

Prior to fluorescence live cell imaging, HeLa cells were cultured for three days on the HCGN platform and were washed with Hank's buffer (Thermo‐Fisher Scientific, Germany). The mitochondrial membranes of live cells were stained for 15 min at 37 °C with MitoBright LT Green (Dojindo Laboratories, Kumamoto, Japan), after which the mitochondrial membrane potential activity was measured. The nuclei of the live cells were counterstained with Hoechst 33342 for 7 min. Mitochondrial superoxide was detected using the fluorescent MitoSox probe (Invitrogen, USA). The cultured human mesenchymal stem cells and MAC‐treated cells were incubated in Hank's buffer with 3 µm MitoSox‐Red for 15 min at 37 °C in a 5% CO_2_ atmosphere. All fluorescence live cell imaging was conducted using a Leica DMi8 confocal microscope. The ImageJ software was used to quantify the images obtained from the hADMSCs cultured in the presence and absence of MAC (*n* = 7).

### Detection and Quantification of Cell Senescence

For the detection of cell senescence, hMSCs were seeded at a concentration of 25 000 cells per well in a 6‐well culture plate. The cultured cells were rinsed with 1× PBS and stained with a *β*‐galactosidase staining kit to evaluate senescence (Cell Signaling Technology, USA) following the manufacturer's instructions. After incubation for 16 h at 37 °C, the stained samples were visualized using an optical microscope (Optinity, Korea Lab Technology, South Korea). To quantify the stained images obtained from the hADMSCs cultured onto each group with each passage, the senescence‐associated *β*‐galactosidase (SA‐*β*‐gal) images (*n* = 5) were analyzed using the ImageJ software.

### Quantitative Real‐Time Reverse Transcription Polymerase Chain Reaction (RT‐qPCR) Analysis for hMSC Multipotency

Total RNA was extracted from mesenchymal stem cells using the RNeasy Mini kit (QIAGEN, Hilden, Germany) for the detection of hMSC multipotency. Complementary DNA (cDNA) was synthesized using Superscript II reverse transcriptase (Invitrogen, Carlsbad, CA, USA). RT‐qPCR amplifications were conducted using SYBR premix EX Taq (Takara, Shiga, Japan) using a StepOnePlus system. Amplifications were conducted using the following protocol: 95 °C for 2 min, followed by 40 cycles at 95 °C for 3 s and 60 °C for 30 s. All RT‐qPCR primer sequences are listed in Table S2 of the Supporting Information. The obtained data were analyzed using the 2^−ΔΔCq^ (Livak) method and relative expressions were calculated using a single housekeeping gene (GAPDH) as reference.

### Flow Cytometry (FACS) Analysis

FACS analysis was conducted using a BD Accuri C6 Plus Flow Cytometer (BD Bioscience, CA, USA). Prior to FACS analysis, the hMSCs (passage 4) were treated with MAC for 90 min at 37 °C to compare the differences in multipotency between the treated group and untreated hMSC groups. The expression of MSC markers (CD 90, CD 105, and CD 73) was used for hMSC multipotency detection. All procedures were conducted according to the manufacturer's instructions and all samples were processed using the BD StemFlow Human MSC analysis kit (BD Bioscience, CA, USA).

### Cytotoxicity and Proliferation Analysis

Cytotoxicity and proliferation testing was conducted using a Cell Counting Kit‐8 (CCK‐8, Dojindo, Kumamoto, Japan). Electrochemically detected HeLa cells and hADMSCs were cultivated on the HCGN platform and then washed with DPBS. After the washing step, 100 µL of prewarmed cultured media and 10 µL of CCK‐8 reagent were mixed and loaded into each chip. These samples were then incubated for an additional 90 min. The absorbance was measured with a microplate reader (Synergy H1 Hybrid Reader, BioTek, VT, USA) at 450 nm.

### Statistical Analysis

All data were presented as the mean ± standard deviation (SD) of three replicates. Multiple comparisons were conducted using one‐way analysis of variance (ANOVA) with Tukey's post hoc test. Pair‐wise comparisons were conducted via an unpaired Student's *t*‐test. All significant differences were presented as *(*p* < 0.05), **(*p* < 0.01), and ***(*p* < 0.001).

## Conflict of Interest

The authors declare no conflict of interest.

## Supporting information

Supporting Information

Supporting Information

## Data Availability

The data that support the findings of this study are available from the corresponding author upon reasonable request.
