# Supplementary material for: Extracellularly Detectable Electrochemical Signals of Living Cells Originate from Metabolic Reactions
Source: Adv Sci (Weinh). 2023 Feb 3;10(9):2207084. doi: 10.1002/advs.202207084 (PMC10037963; doi:10.1002/advs.202207084)
Supplement: Supplementary file 1 — Supporting Information [file ADVS-10-2207084-s002.pdf]

Supporting Information

Extracellularly detectable electrochemical signals of living cells originate from metabolic reactions

*Kyeong-Mo Koo, Chang-Dae Kim, Huijung Kim, Yeon-Woo Cho, Intan Rosalina Suhito, Tae-Hyung Kim**

K. -M. Koo, C. -D. Kim, H. Kim, Y. -W. Cho, I. R. Suhito, and Prof. T. –H. Kim
School of Integrative Engineering
Chung-Ang University, Seoul 06974, Republic of Korea
Fax: (+82) 2-820-5469
Email: thkim0512@cau.ac.kr

I. R. Suhito
Department of Biomedical Engineering
National University of Singapore, 117583, Singapore

Keywords: Electrochemical detection, Metabolic reaction, Live cell sensing, Drug screening, Stem cell senescence

Contents:

Figures

Figure S1 | Characterisation of the HCGN platform

Figure S2 | DPV detection from HeLa cells with varying $HAuCl_4$ electrodeposition times

Figure S3 | DPV signals for verification of the stability and reproducibility of the HCGN platform

Figure S4 | Electrochemical signals of MMPs at various conditions

Figure S5 | Electrochemical signals of extracted mitochondria

Figure S6 | DPV signals from HeLa cells treated with mitochondrial complex inhibitors

Figure S7 | ATP assay and final cell counting results after Rot/AA and oligomycin treatment

Figure S8 | CCK-8 results of HeLa cells treated with various mitochondrial complex inhibitors

Figure S9 | MitoBright staining after FCCP treatment

Figure S10 | Optimisation of treatment time for amplification of redox signals on the HCGN platform and DPV signals of HeLa cells at various times

Figure S11 | DPV voltammogram of cancer cells and stem cells treated with MAC

Figure S12 | MitoBright and ATP-Red 1 images of cells treated with various concentrations of CPI-613

Figure S13 | Bright-field images of hADMSCs stained with SA-β-gal

Figure S14 | Flow cytometry results of hADMSCs at several passages and comparison between CTL (–MAC) and MAC-treated cells (+MAC)

Figure S15 | DPV signals of hADMSCs under various growth medium compositions and comparison between CTL (–MAC) and MAC-treated cells (+MAC)

Tables

Table S1 | Electrochemical detection for various types of cells and comparison between CTL (–MAC) and MAC-treated cells (+MAC)

Table S2 | Primers used in this study

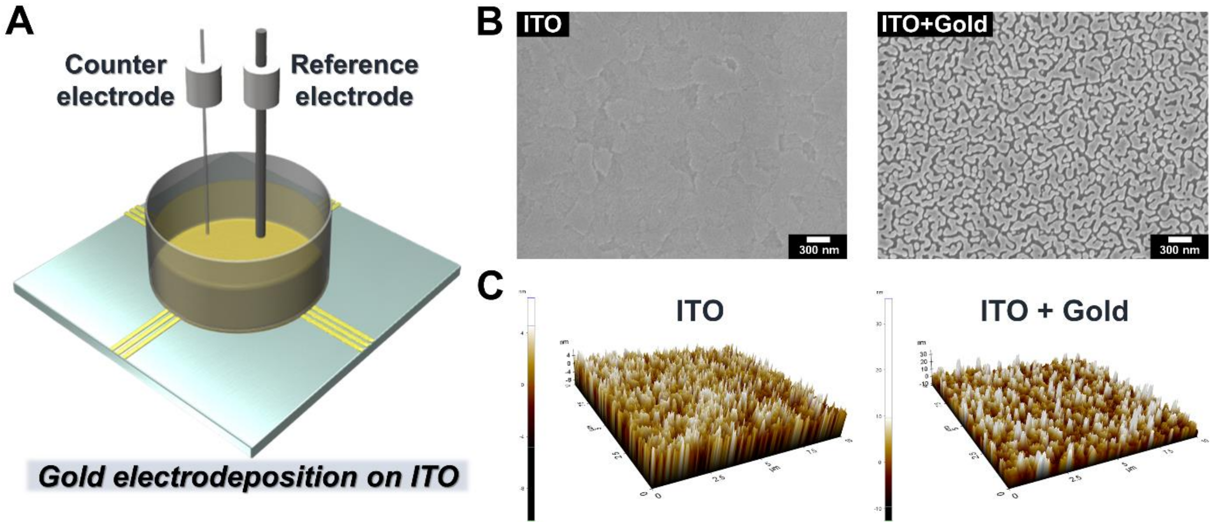

Figure S1. A) Schematic illustration of $HAuCl_4$ electrodeposition on indium tin oxide (ITO) glass. **B)** FE-SEM visualisation of the ITO and highly conductive gold nanostructure (HCGN) platform. **C)** AFM analysis of the ITO and HCGN platform.

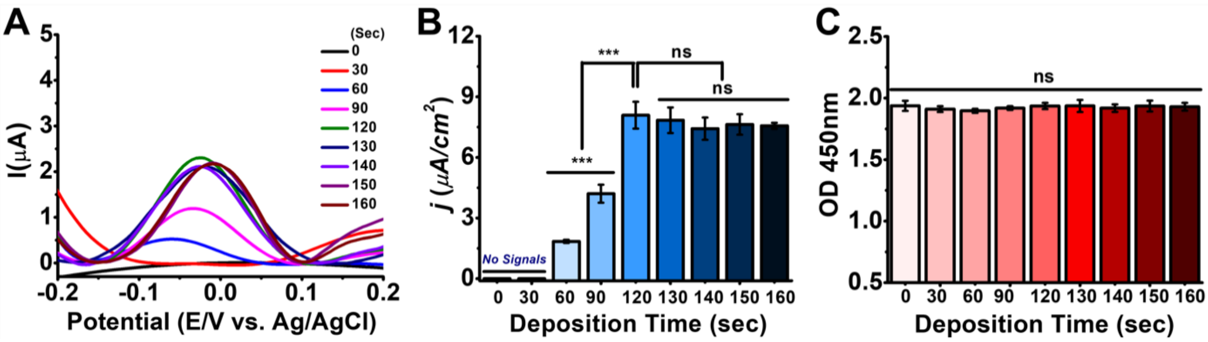

Figure S2. A) DPV signals (E_p = –0.05 V) detected from HeLa cells cultured on substrates with varying $HAuCl_4$ electrodeposition times. **B)** Current densities calculated from (A) presented as a bar graph (j represents the current density, *** $p < 0.001$, n = 3). **C)** CCK-8 results of HeLa cells cultured on the HCGN platform at various deposition times, respectively.

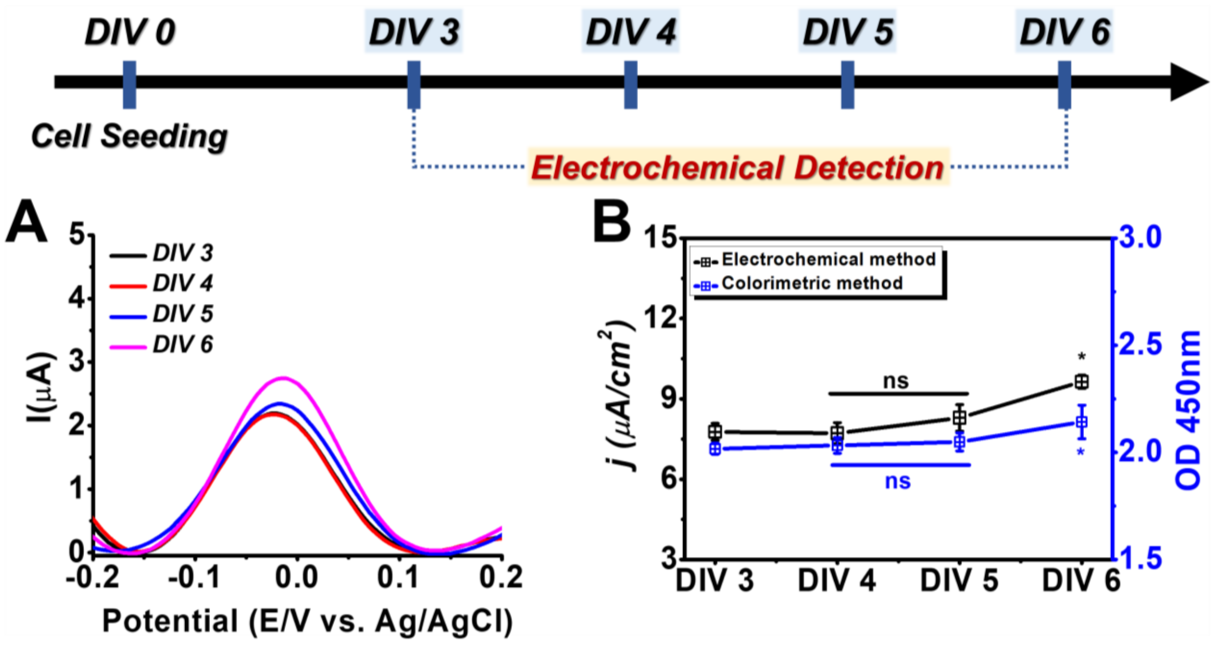

Figure S3. A) DPV results from DIV 3 to DIV 6. **B)** Calculated current densities and CCK-8 results measured in (A) are shown as a double-y graph (* $p < 0.05$, $n = 3$).

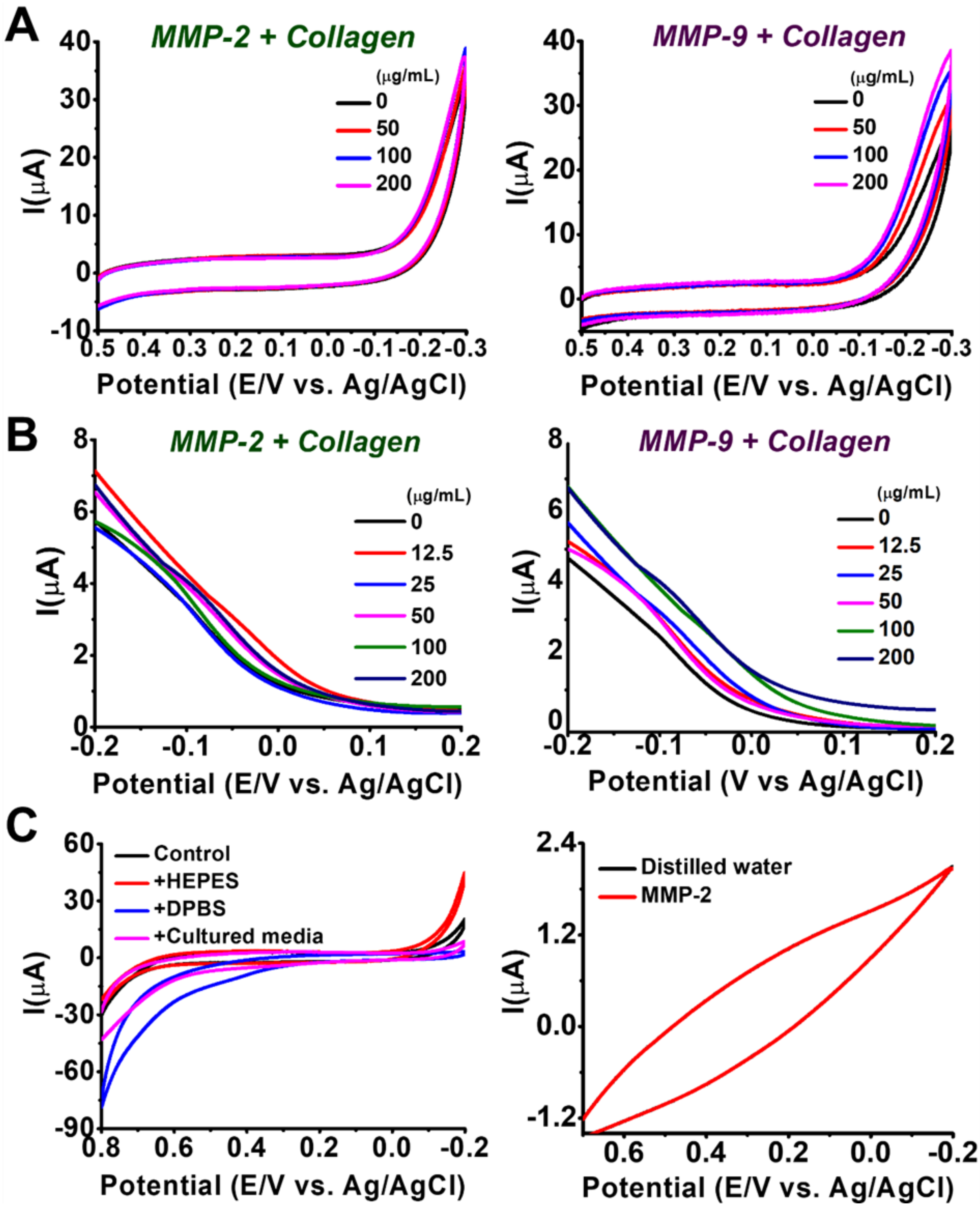

Figure S4. (A) Cyclic voltammetry (CV) signals detected from MMP-2 (left panel) and MMP-9 (right panel) loaded on collagen-coated platforms (for 1 h) at various concentrations. **B)** Differential pulse voltammetry (DPV) detection from MMP-2 (left panel) and MMP-9 (right panel) at varying concentrations ranging from 0 to 200 ng/mL. **C)** CV signal of various solvents including 1X PBS (no treatment), HEPES, DPBS, and culture media (10% FBS + supplemented DMEM). CV signals of MMP-2 diluted in distilled water and no MMP-2 (overlapped).

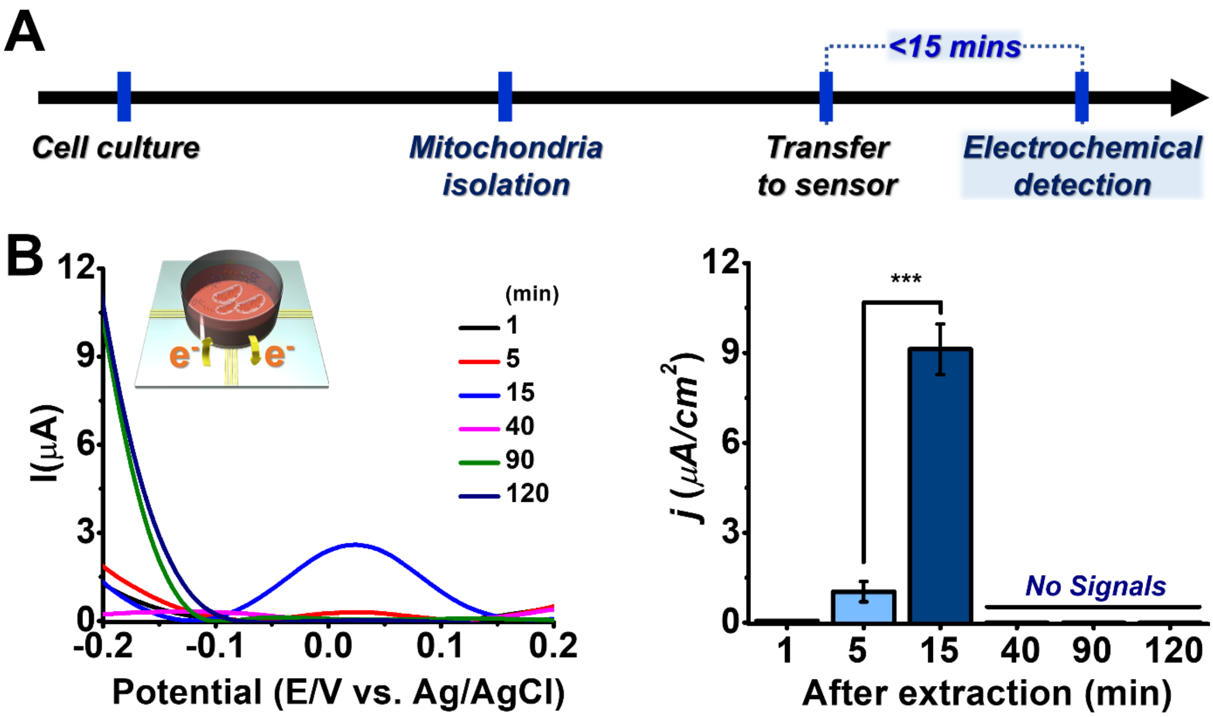

Figure S5. A) Schematic illustrations showing the process of mitochondrial isolation and electrochemical detection. **B)** Detectable time optimisation for electrochemical signal measurement at various times ranging from 1 to 120 mins. The current density calculated from the DPV graph (left panel) is shown as a bar graph. (*** $p < 0.001$, $n = 4$).

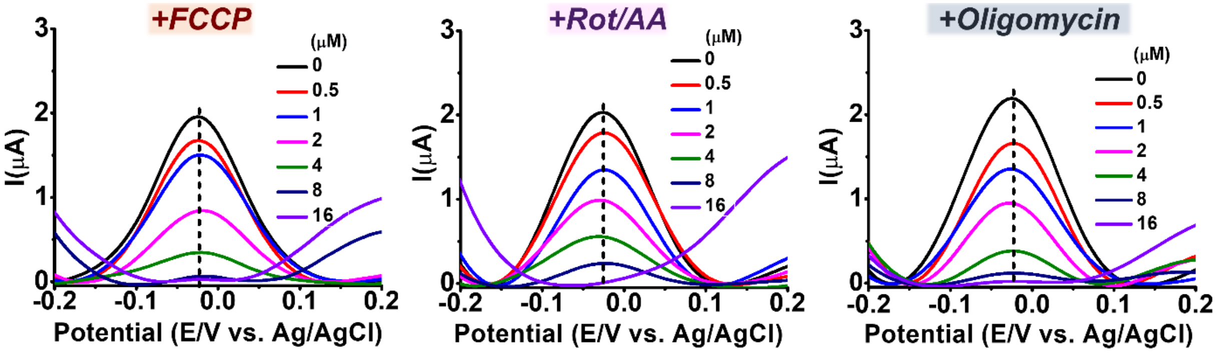

Figure S6. DPV signals from HeLa cells treated with ROT & AA, oligomycin, and FCCP for 24 h at various concentrations ranging from 0 to 16 μM.

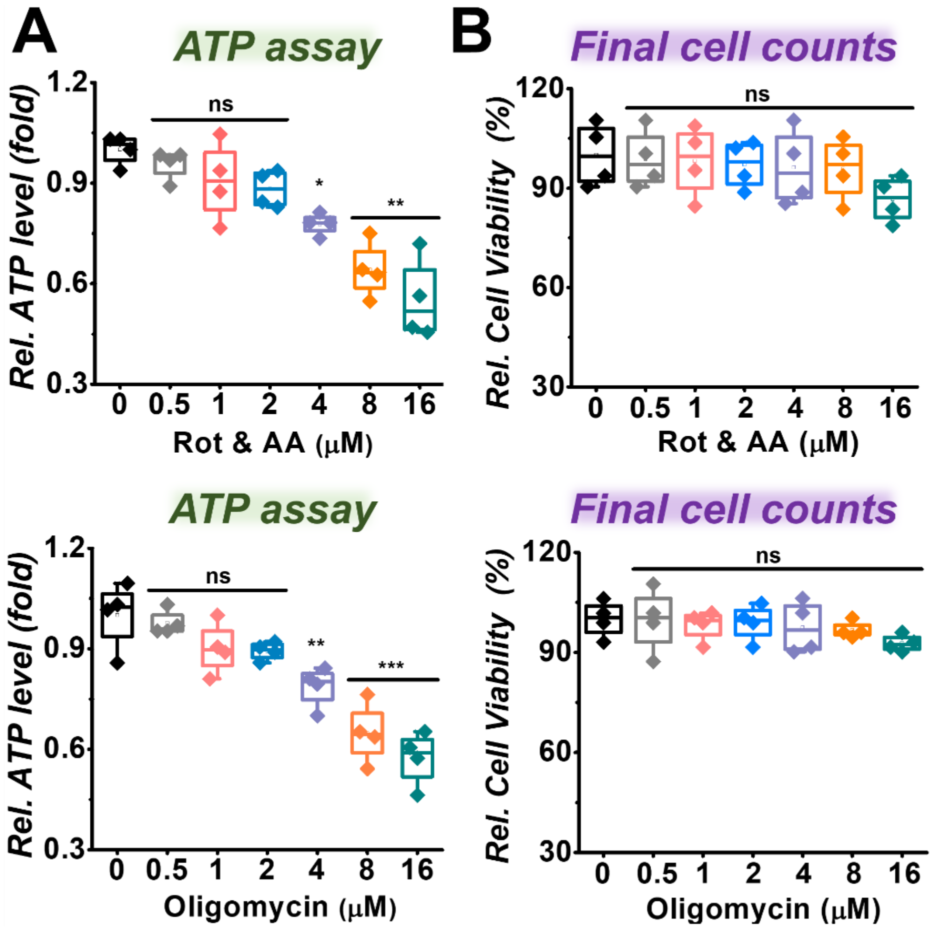

Figure S7. A) Box plot of the calculated ATP level percentages from HeLa cells exposed to different concentrations of Rot/AA and oligomycin (* $p < 0.05$, ** $p < 0.01$, *** $p < 0.001$, $n = 4$). **B)** Final cell counting after DPV detection and treatment with Rot/AA and oligomycin for 24 h (*** $p < 0.001$, $n = 4$).

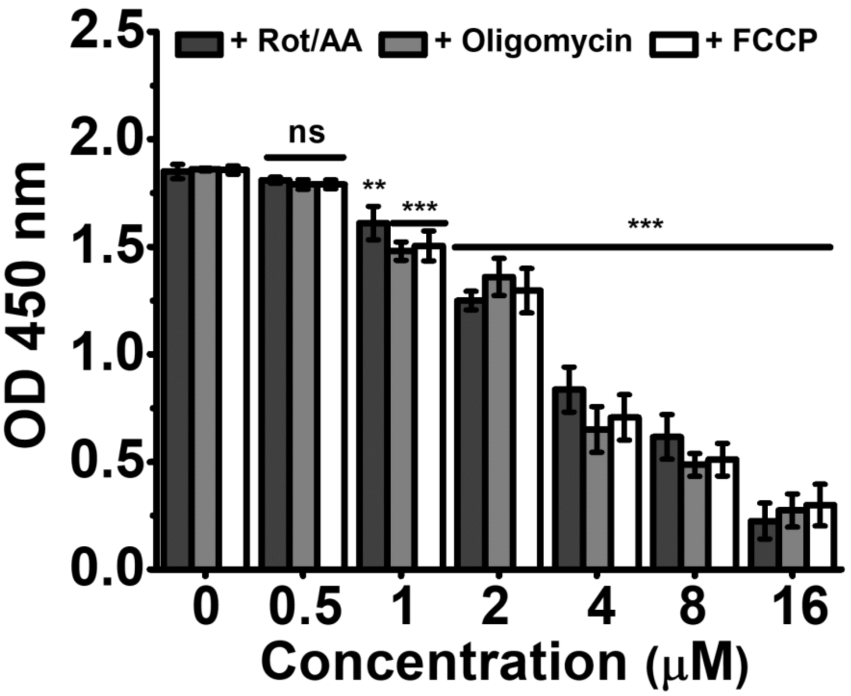

Figure S8. CCK-8 results of HeLa cells treated with mitochondrial complex inhibitors at various concentrations for 24 h.

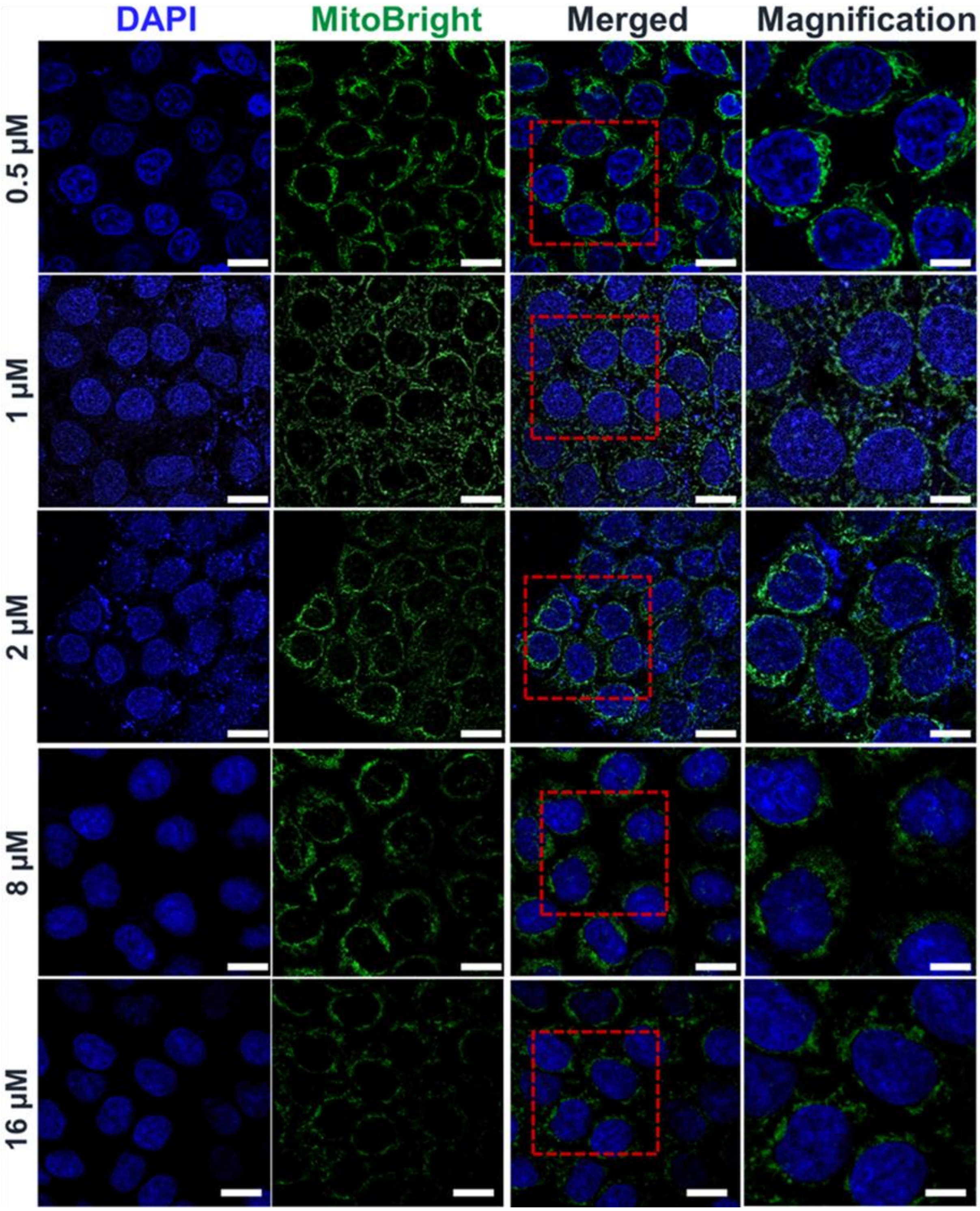

Figure S9. Confocal images of HeLa cells treated with FCCP at 0.5, 1, 2, 8, 16 μM for 24 h and stained with MitoBright Green (0.1 μmol/L), with nuclear counterstaining using Hoechst 33342 (scale bar: 8 μm), and magnified images (scale bar: 3 μm).

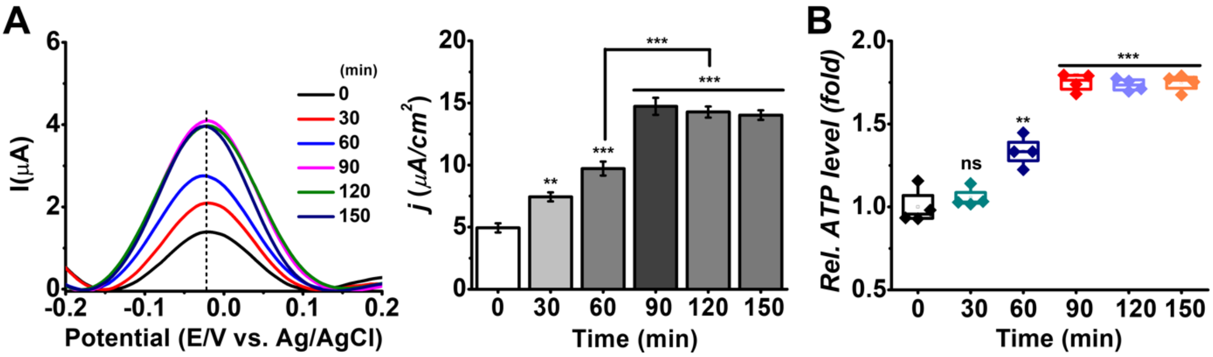

Figure S10. A) Optimisation of treatment time for amplification of redox signals on the HCGN platform and DPV signals of HeLa at various times ranging from 0 to 150 mins. Current densities based on the data presented in the DPV graph (left panel), (** $p < 0.01$, *** $p < 0.001$, $n = 5$). **B)** Quantitative analysis of ATP levels of HeLa cells treated with MAC at various concentrations; the data were normalised to the control (0 min) (** $p < 0.01$, *** $p < 0.001$, $n = 4$).

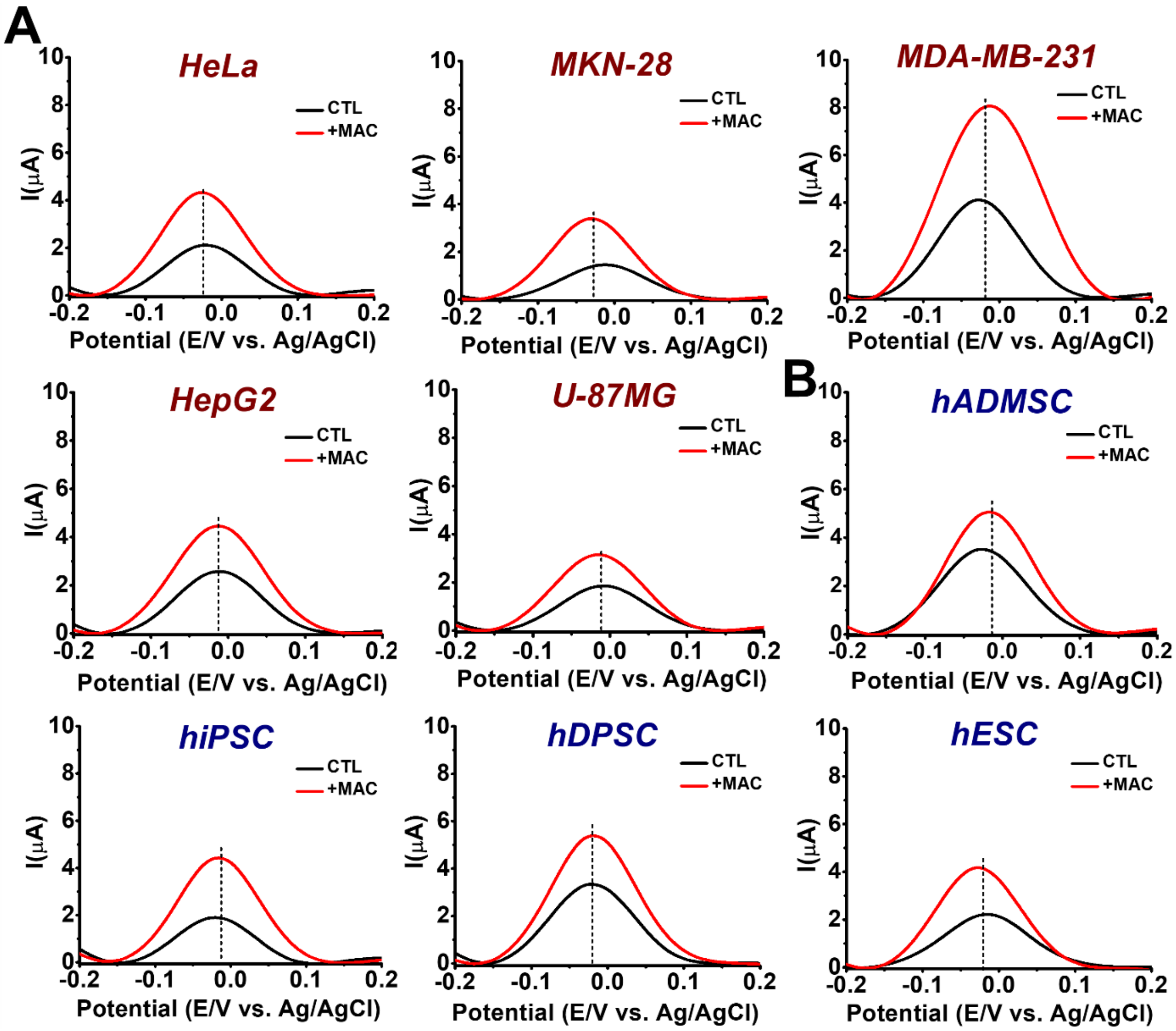

Figure S11. A-B), DPV voltammogram of cancer cells and stem cells treated with MAC for 90 min cultured on the HCGN platform.

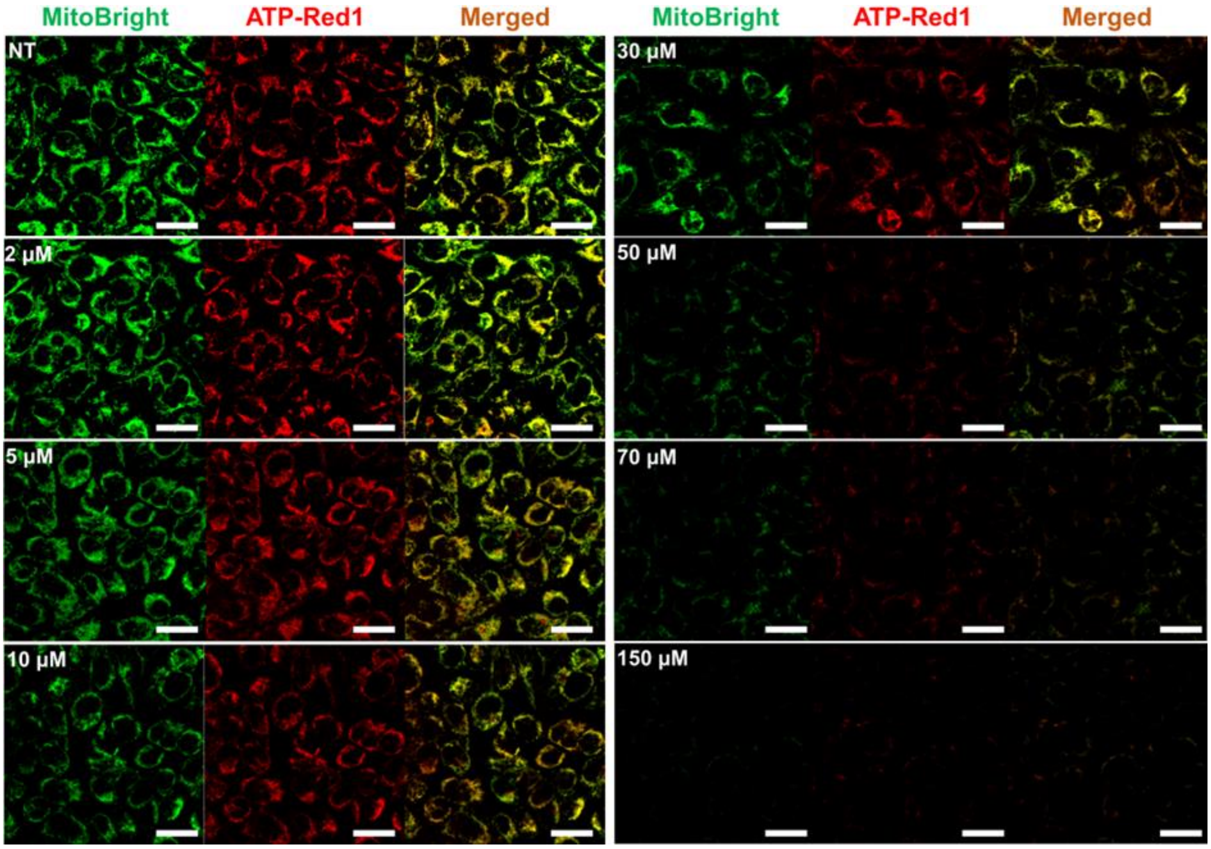

Figure S12. Live HeLa cells were stained with MitoBright (0.1 µmol/L) and ATP-Red 1 (5 µM) and treated with various concentrations (2, 5, 10, 50, 70, and 150 µM) of CPI-613 for 24 h (scale bar: 8 µm).

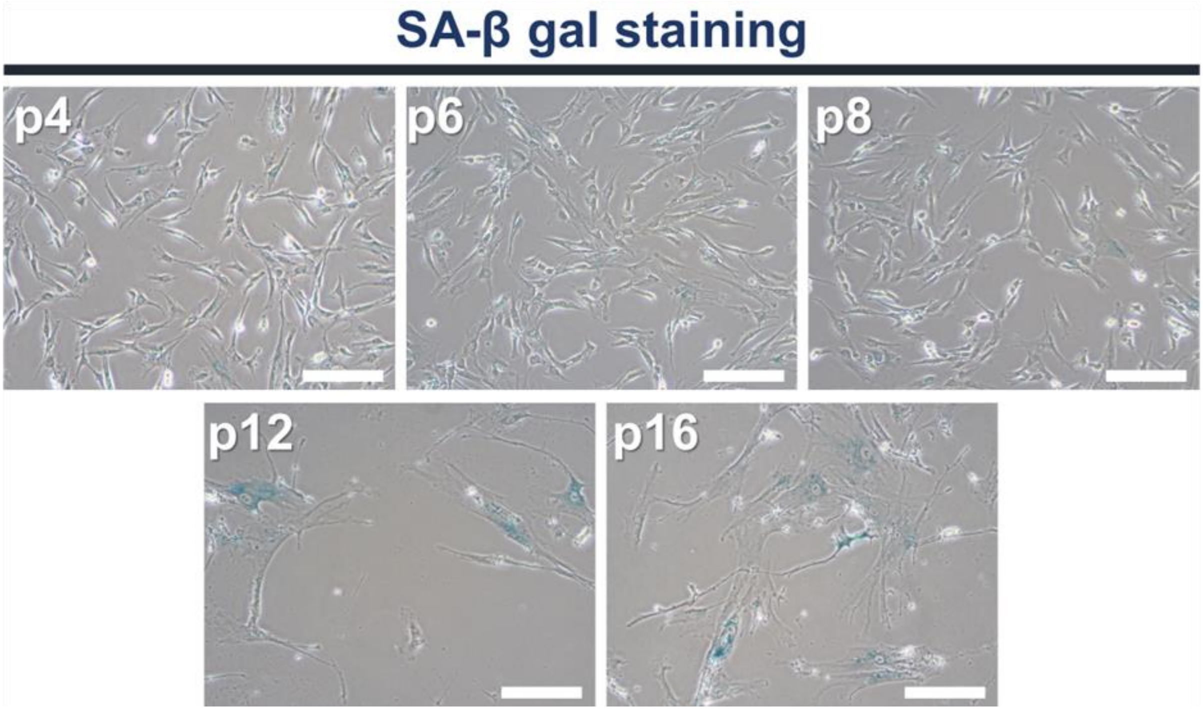

Figure S13. Representative bright-field images of hADMSCs (P4, P6, P8, P12, and P16) stained with senescence-associated beta-galactosidase (SA-β-gal; blue) at pH 6.0 (scale bar: 250 µm).

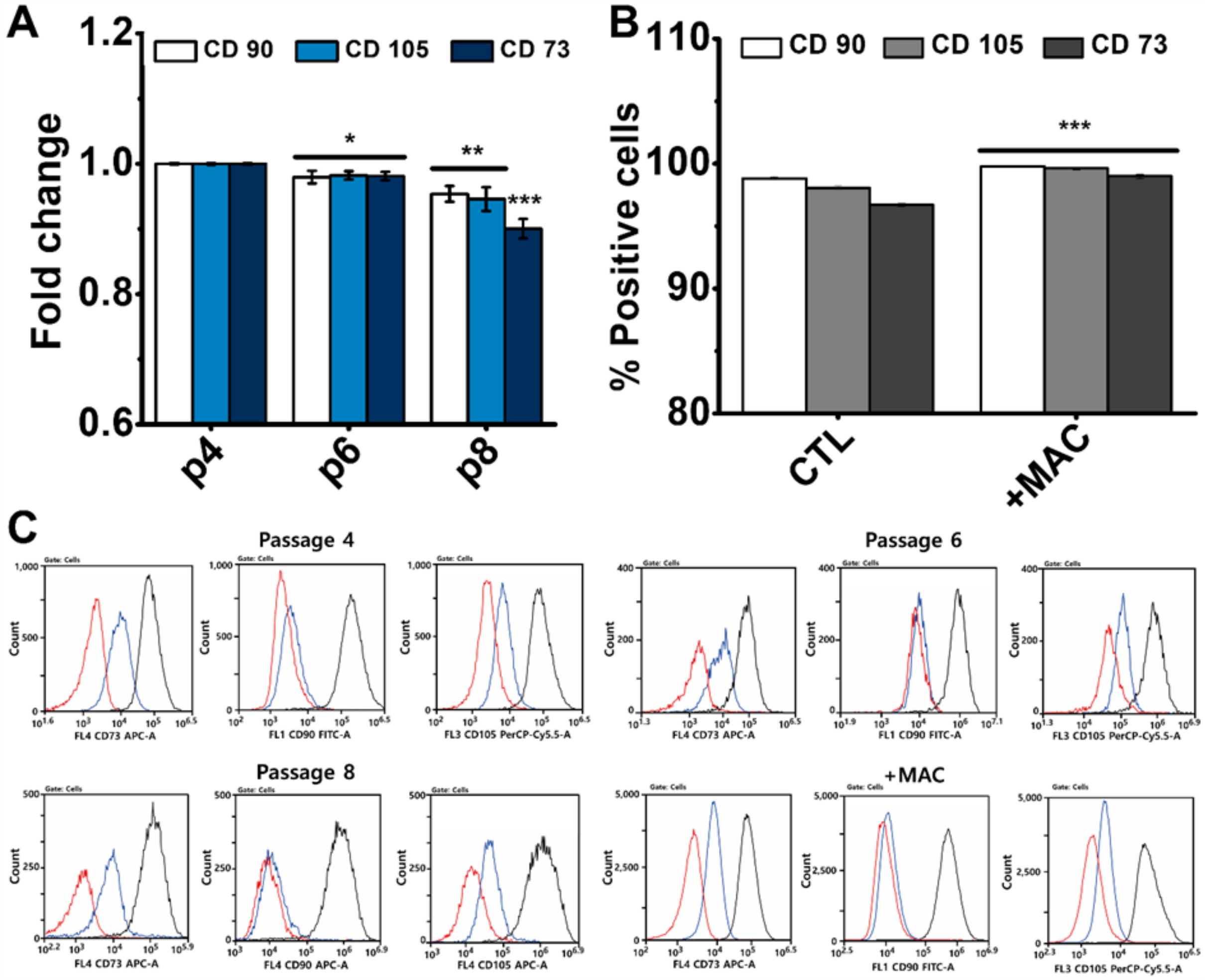

Figure S14. Protein expression results of hADMSCs at several passages; the data were normalised to the control (P4) (* p < 0.05, ** p < 0.01, *** p < 0.001, n = 3). **A)** Flow cytometry results of hADMSCs cultured on the HCGN platform and comparison between CTL (–MAC) and MAC-treated cells (+MAC); the data were normalised to the control (passage 4) (*** p < 0.001, n = 3). **B)** Flow cytometry results of hADMSCs cultured on the HCGN platform and comparison between CTL (–MAC) and MAC-treated cells (+MAC); the data were normalised to the control (P4) (*** p < 0.001, n = 3). **C)** Flow cytometry analysis of CD73, CD90, and CD105 expression of hADMSCs grown on the HCGN platform at several passages.

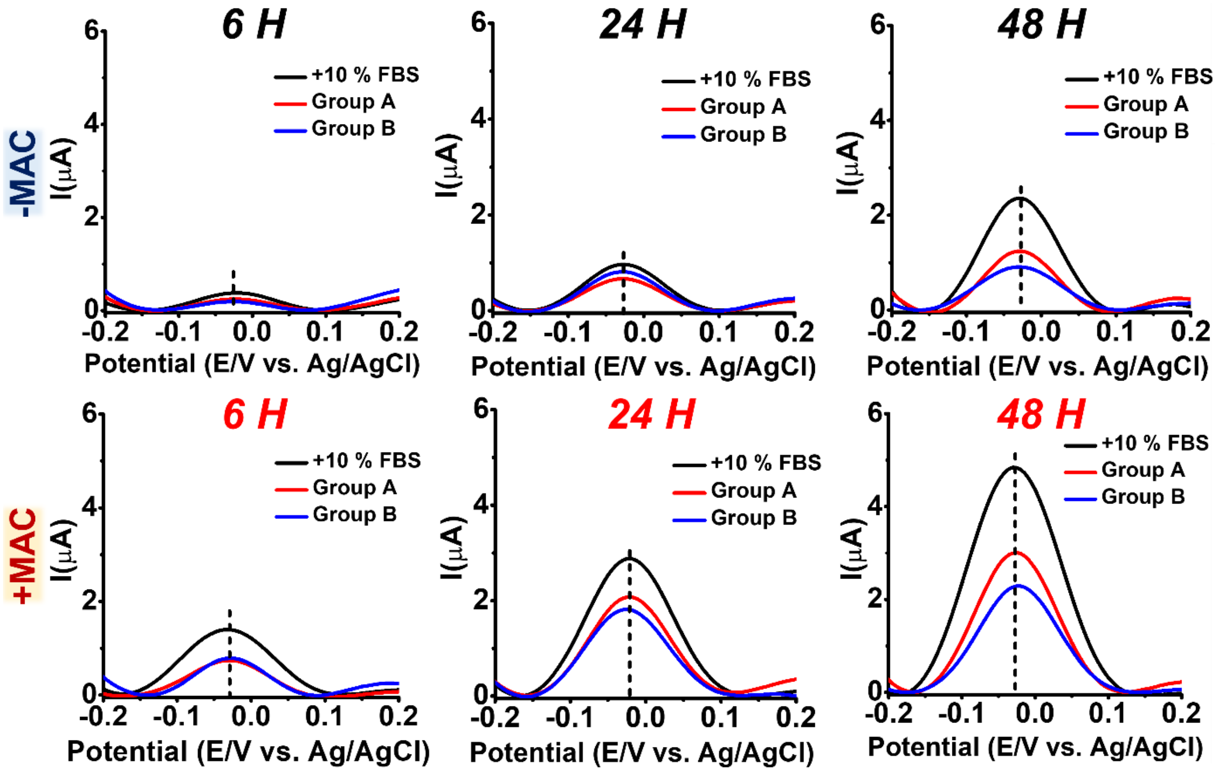

Figure S15. DPV signals of hADMSCs under various growth medium compositions with and without MAC treated for 6, 24, and 48 h to confirm cell proliferation.

Table S1 | Electrochemical detection for various types of cells without MAC (CTL) and with MAC (+MAC)

	Control (CTL)			+MAC		
**Cell type**	**Final cells (cells/chip)**	**I_p (µA/chip)**	**Single cell unit (µA/cell)**	**Final cells (cells/chip)**	**I_p (µA/chip)**	**Single cell unit (µA/cell)**
**HepG2**	24,163	$2.49E^{-06}$	$1.03E^{-10}$	24,625	$4.38E^{-06}$	$1.78E^{-10}$
**U-87 MG**	20,625	$1.89E^{-06}$	$9.23E^{-11}$	21,100	$3.15E^{-06}$	$1.54E^{-10}$
**HeLa**	23,875	$2.08E^{-06}$	$8.71E^{-11}$	24,500	$4.32E^{-06}$	$1.77E^{-10}$
**MKN-28**	20,500	$1.46E^{-06}$	$7.1E^{-11}$	20,625	$3.35E^{-06}$	$1.63E^{-10}$
**MDA-MB-231**	33,500	$4.06E^{-06}$	$1.21E^{-10}$	36,875	$8.18E^{-06}$	$2.23E^{-10}$
**hESC**	39,363	$2.2E^{-06}$	$5.68E^{-11}$	38,375	$4.07E^{-06}$	$1.08E^{-10}$
**hiPSC**	38,475	$1.87E^{-06}$	$4.86E^{-11}$	43,875	$4.43E^{-06}$	$1.01E^{-10}$
**hADMSC**	292,50	$3.38E^{-06}$	$1.19E^{-10}$	24,125	$4.99E^{-06}$	$2.09E^{-10}$
**hDPSC**	33,625	$3.34E^{-06}$	$1.02E^{-10}$	34,500	$5.41E^{-06}$	$1.66E^{-10}$

Table S2 | Primers used in this study

Gene	Forward Sequence (5' to 3')	Reverse Sequence (5' to 3')
**GAPDH**	CTGGCGCTGAGTACGTCG	TTGACAAAGTGGTCGTTG
**CD44**	GGCTTTCAATAGCACCTTGC	ACACCCCTGTGTTGTTTGCT
**CD73**	CAGTACCAGGGCACTATCTGG	AGTGGCCCCTTTGCTTTAAT
**CD90**	ATGAACCTGGCCATCAGCA	GTGTGCTCAGGCACCCC
**CDH11**	GGTCTGGAACCAGTTCTTCG	TCTCGATCCAACGTCTTGGT
